# Imaging in the postpartum period

**DOI:** 10.1016/j.ejro.2026.100789

**Published:** 2026-07-01

**Authors:** Girija Agarwal, Nishat Bharwani, Sadia Raheez Qamar, Marisa Taylor-Clarke, Elika Kashef, Raffaella Basilico, Elizabeth Dick

**Affiliations:** a Department of Radiology, Imperial College Healthcare Trust, LondonW2 1NY UK; b Department of Surgery and Cancer, Imperial College London, UK; c Department of Medical Imaging, Sunnybrook Health Science Centre, University of Toronto, Toronto, ON M4N 3M5, Canada; d Department of Obstetrics and Gynaecology, Imperial College Healthcare Trust, LondonW2 1NY UK; e Department of Medical, Oral and Biotechnological Sciences, University G. D′Annunzio Chieti-Pescara, Chieti, Italy

**Keywords:** Postpartum imaging findings, Retained products of conception, Postpartum haemorrhage, Endometritis, Postpartum complications

## Abstract

The postpartum period is characterised by significant physiological and anatomical changes that may mimic pathology on imaging. Exposure to puerperal imaging varies between healthcare systems and may depend on whether postnatal ultrasound is performed primarily by radiologists or obstetricians/gynaecologists. Differentiating normal postpartum appearances from true complications can therefore be challenging, particularly when clinical presentations are non-specific. This review summarises normal and abnormal postpartum imaging findings and describes a structured checklist to systematically evaluate the pelvic organs.

## Highlights

• Summarises normal and abnormal postpartum imaging appearances.
• Provides a structured checklist for systematic pelvic organ evaluation.
• Addresses key diagnostic challenges in puerperal imaging interpretation.
• Enhances radiologists’ confidence in postpartum image assessment.

## Graphical Abstract

**Figure:**
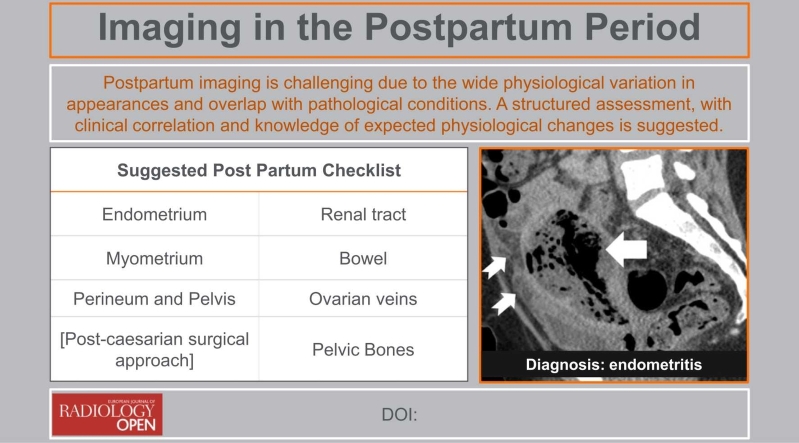

**Summary**

Postpartum imaging is challenging due to the wide physiological variation in appearances and the overlap with pathological conditions. A structured, centre-outward assessment combined with clinical correlation and knowledge of expected postoperative changes can substantially improve diagnostic accuracy.

**Key Learning Points**

• Normal postpartum imaging findings often mimic pathology.
• Correlation with clinical presentation and time postpartum is crucial.
• Ultrasound is first line diagnostic modality, but CT/MRI have important roles when findings are equivocal, or complications are suspected.

## 1 Introduction

The postpartum period, also known as the puerperium, represents the 6–8 weeks following pregnancy and birth in which the body undergoes dynamic physiological and anatomical changes [1]. During this period, the uterus involutes and pelvic structures return to their non-gravid state. Radiologists’ exposure to puerperal imaging varies between healthcare systems and local clinical practice. In some settings, postnatal ultrasound is performed primarily by obstetricians/ gynaecologists, with radiologists more commonly involved when CT or MRI is required. In addition, imaging findings during the puerperium can be highly variable and often overlap with pathological conditions which can make interpretation challenging.

This review provides an overview of imaging in the postpartum period, highlighting normal findings, common complications, and a systematic approach to evaluate the pelvic structures. Dedicated post-caesarean surgical complications, such as uterine niche defects or bladder-flap haematomas are beyond the scope of this review. However, general puerperal complications such as postpartum haemorrhage, endometritis or retained products of conception may occur after either vaginal or caesarean birth and are therefore discussed where relevant.

### 1.1 Learning objectives

1. To recognise normal postpartum appearances on ultrasound, CT, and MRI.
2. To improve diagnostic confidence in identifying abnormal features and differentiating them from physiological changes.
3. To develop a structured checklist when evaluating postpartum imaging.

### 1.2 Diagnostic challenges in postpartum imaging

Postpartum imaging presents several diagnostic challenges. Firstly, radiologists’ exposure to puerperal imaging is variable. Although postpartum CT and MRI are generally interpreted by radiologists, first-line postnatal ultrasound may be performed by obstetricians or gynaecologists. As a result, radiologists may encounter postpartum imaging with variable frequency and be asked to interpret cross-sectional imaging without direct access to the initial ultrasound findings [1].

Secondly, there is considerable overlap of normal and abnormal appearances. For example: i) Echogenic endometrial material can be seen in up to 50% of asymptomatic women in the first seven days after birth but can also represent retained products of conception (RPOC) or endometritis [2]. ii) Uterine vascularity varies widely postpartum, and increased Doppler vascularity may be seen in both physiological postpartum change as well as retained products of conception (RPOC), making differentiation challenging [2]. (iii) Gas in the endometrial cavity may persist for up to three weeks postpartum but can also reflect endometritis [3], [4].

In addition to the imaging findings themselves, the clinical context can also add to the challenge. Patients often present with non-specific symptoms such as pain, fever, or bleeding. Furthermore, radiologists may only be provided with fragmented clinical context as obstetricians or gynaecologists frequently perform the initial ultrasound, with radiology consulted later for cross-sectional imaging (CT/MRI) which limits correlation with early findings.

### 1.3 Definitions

The postpartum period is defined as the 6–8 weeks following birth of the foetus and placenta. It may also follow a spontaneous or elective abortion. Importantly, up to 75% of perinatal maternal deaths occur within this period, underscoring the importance of accurate diagnosis and timely intervention when complications arise [5]. In the UK from 2021 to 2023, 257 women died during or up to six weeks after pregnancy among 2004,184 women giving birth, representing a maternal death rate of 12.82 women per 100,000 women giving birth [6].

### 1.4 Imaging approach: a systematic checklist

To ensure consistent evaluation, we describe a structured approach, beginning centrally with the endometrial cavity and extending outwards. The following sections outline key imaging findings for each region, including normal variations and common complications.

Suggested systematic checklist for evaluating postpartum imaging:

1. Endometrium
2. Myometrium
3. Perineum and Pelvis
4. Post-caesarean surgical approach
5. Renal tract
6. Bowel
7. Ovarian veins
8. Pelvic Bones (MRI)

### 1.5 Normal postpartum imaging appearances

The uterus remains enlarged in the early postpartum period and gradually involutes over the puerperium. The endometrial cavity may contain fluid, blood products, clot, echogenic debris, or small foci of gas, particularly early postpartum [2], [3], [4]. The myometrium may appear bulky and relatively hypervascular, reflecting physiological involution and increased uterine blood flow [7].

Beyond the uterus, physiological dilatation of the ovarian veins and mild hydroureter may persist transiently after pregnancy [8]. Rectus divarication may be seen in the abdominal wall and may be mistaken for a fascial defect [9]. On MRI, pubic symphyseal bone marrow oedema may be seen in the early postpartum period and should be interpreted with the clinical presentation [10]

### 1.6 Endometrium and endometrial cavity

#### 1.6.1 Normal physiology and anatomy of endometrium

The decidua which is derived from the endometrium, provides support for implantation and placenta formation. The placenta comprises foetal and maternal components: foetal vessels in the chorionic villi are bathed in maternal blood within the intervillous spaces supplied by uterine arteries [11].

**Fig. 1:**
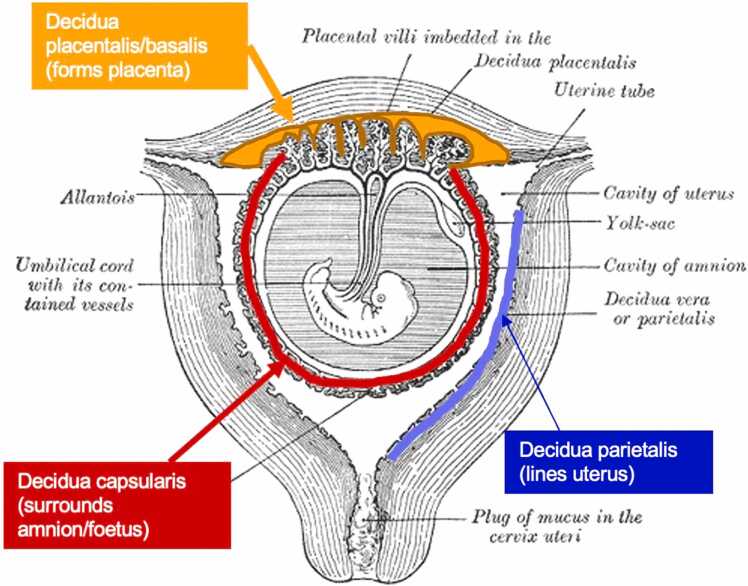

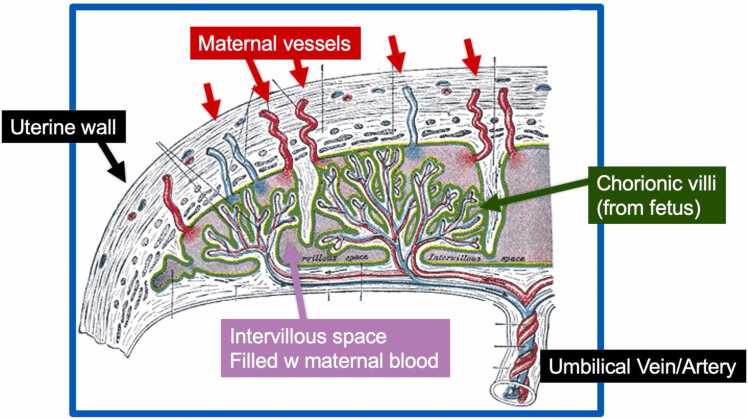
**a. Normal anatomy of the gravid uterus in the fourth week of gestation. The decidua, a matrix which forms from endometrium every month can be shed as menstrual blood or supports embryo implantation & placenta formation**. Image credit: Adapted from sectional plan of the gravid uterus in the third and fourth month (modified from Wagner) from Carter HV, in Gray H, Lewis WH, editors. *Anatomy of the Human Body*. 20th ed. Philadelphia: Lea & Febiger; 1918 [13]. . **b. Normal anatomy of the placenta with both foetal and maternal components.** Image credit: Adapted scheme of placental circulation from Carter HV, in Gray H, Lewis WH, editors. *Anatomy of the Human Body*. 20th ed. Philadelphia: Lea & Febiger; 1918 [14]. .

After birth, the placenta detaches and is expelled. A subplacental haematoma commonly forms, and myometrial contractions constrict blood vessels to prevent haemorrhage. The endometrial cavity can contain fluid, clots, or gas, all of which may be normal. Vaginal discharge, or lochia, consisting of blood, mucus, and decidual debris, persists for up to six weeks [12]. See Fig. 1 [13], [14].

#### 1.6.2 Complications

The common complications that occur within the endometrial cavity include postpartum haemorrhage, RPOC and endometritis. Each may occur in isolation but can also commonly co-present.

#### 1.6.3 Postpartum haemorrhage

Postpartum haemorrhage (PPH) can be either primary or secondary. Primary PPH is defined as blood loss of over 500 ml from the genital tract occurring within 24 h after birth. Secondary PPH is deﬁned as abnormal or excessive bleeding from the genital tract occurring between 24 h and 12 weeks postnatally [15].

Common causes of PPH are frequently remembered as the “four Ts.” Namely 1) Tone: this refers to uterine atony for which the risk factors include multiple pregnancies and prolonged labour. 2) Trauma: risk factors for this are episiotomy and perineal tears. 3) Thrombin: referring to coagulopathies including those related to pre-eclampsia. 4) Tissue: referring to retained placental tissue or abnormally invasive placenta (placenta accreta) [15].

Imaging appearances of postpartum haemorrhage depends on the underlying cause, Uterine atony is usually a clinical diagnosis however, when bleeding is persistent or an alternative cause is suspected CT can be useful. In uterine atony, CT may demonstrate an enlarged postpartum uterus containing intrauterine haematoma with contrast extravasation into the uterine cavity. Arterial bleeding is usually best demonstrated on arterial phase imaging, whereas small venous oozing may be more conspicuous on delayed phase imaging. CT can also identify uterine artery pseudoaneurysm, arteriovenous malformation or retained products of conception as well as extra uterine causes of PPH, including perineal/genital haematomas, broad ligament or rectus sheath haematomas [16].

#### 1.6.4 Uterine atony

**Fig. 2:**
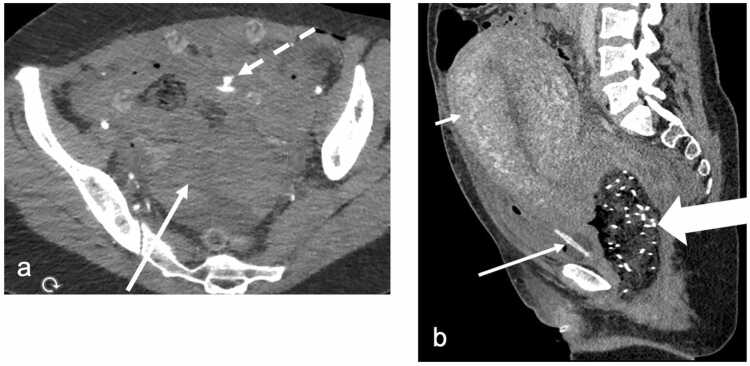
a) Arterial phase CT in patient with postpartum haemorrhage. CT demonstrates large volume haemoperitoneum (long arrow) and active bleeding (dashed arrow). The patient was successfully treated with internal iliac embolization. DIAGNOSIS: postpartum haemorrhage. b) Post partum haemorrhage due to vaginal laceration treated with vaginal packing (block arrow). No active bleeding, packing is dry. Catheter in bladder (long arrow) and normal vascular myometrium (short arrow) noted. DIAGNOSIS: postpartum haemorrhage.

**Fig. 3:**
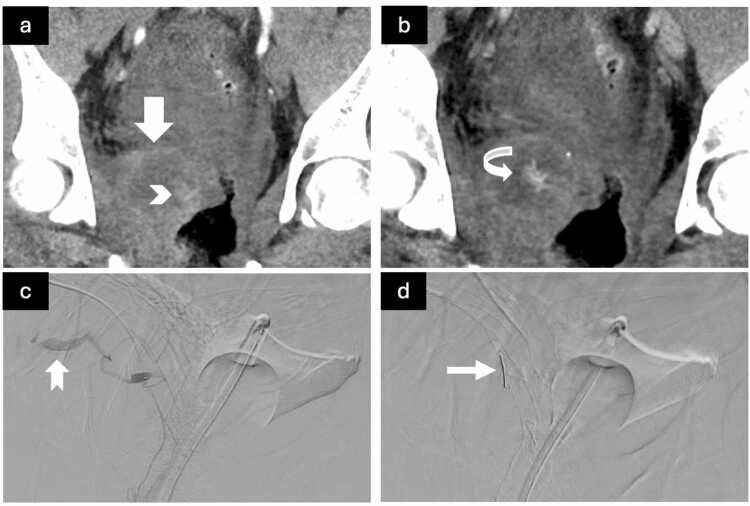
30year – old female with intrapartum 2nd degree vaginal tear repaired in OR. 8 h postpartum patient dropped hemoglobin. Coronal CT pelvis (a) arterial phase (b) delayed phase shows a large pelvic hematoma along the right cervicovaginal wall (bold arrow) with faint contrast extravasation on arterial phase (chevron) and contrast pooling on the delayed phase (curved arrow). Digital subtraction angiography (c) confirms active hemorrhage from vaginal branch of the right uterine artery (notched arrow) (d) successfully embolized (thin arrow). DIAGNOSIS: postpartum haemorrhage.

**Fig. 4:**
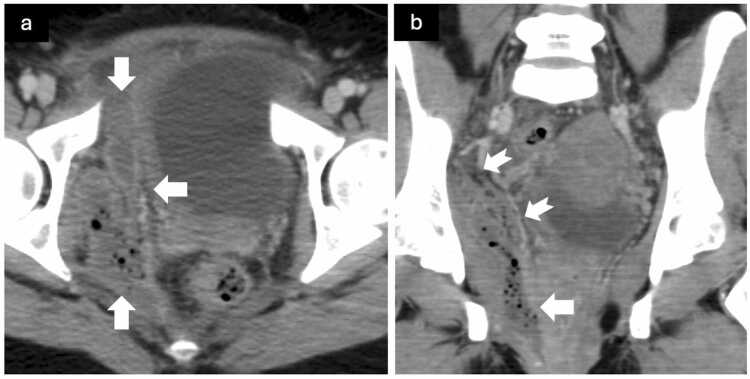
30year – old female with prolonged labor and instrumented vaginal birth presenting 2 weeks postpartum with sepsis. Contrast enhanced CT pelvis (a) axial and (b) coronal images show a right paravaginal mixed density complex fluid and gas containing multiloculated collection (bold arrows) consistent with infected vaginal hematoma with supralevator extension (notched arrows). DIAGNOSIS: postpartum haemorrhage and secondary infection.

Uterine atony is a clinical diagnosis defined as inadequate or absent uterine contraction after birth, resulting in failure of myometrial fibres to compress the uterine vasculature. It is the most common cause of primary PPH, accounting for 70–90% of cases [17]. The pathophysiology involves impaired myometrial contractility due to several mechanisms, including uterine overdistension (multiple gestation, polyhydramnios, macrosomia), prolonged or precipitous labour, chorioamnionitis, uterine exhaustion, and intrinsic myometrial dysfunction such as receptor desensitisation to oxytocin or underlying myometrial pathology (e.g., fibroids) [18], [19]. Uterine atony is usually diagnosed clinically and managed with uterotonics, bimanual compression, and uterine tamponade; imaging is not routinely required. However, contrast-enhanced CT becomes essential when haemorrhage persists despite appropriate medical therapy, when the source of bleeding is uncertain, or when there is concern for alternative diagnosis [18], [20]. CT is particularly valuable to identify active extravasation, pelvic haematomas, and vascular injury patterns. Thus, while uterine atony is a clinical diagnosis, CT plays a critical role in evaluating refractory or unexplained PPH and in guiding urgent interventional or surgical management (see Fig. 2, Fig. 3, Fig. 4).

#### 1.6.5 Uterine artery pseudoaneurysm

A uterine artery pseudoaneurysm (UAP) is a rare but potentially life-threatening vascular lesion that arises secondary to arterial laceration or injury - for example during caesarean section, uterine curettage, operative vaginal birth, or less commonly after an uncomplicated vaginal birth - and fails to seal completely, resulting in a contained haematoma with persistent communication to the uterine artery lumen [21]. Clinically, UAP most often manifests in the delayed postpartum period (i.e., days to weeks after birth), as a cause of secondary PPH, sometimes accompanied by lower abdominal pain, flank/back pain, or even haemoperitoneum without vaginal bleeding (particularly if rupture is intraperitoneal) [22]. Because of non-specific presentations and rare incidence, the diagnosis may be delayed or initially mistaken for more common entities such as RPOC, subinvolution, endometritis, uterine atony, or surgical-site haematoma [23], [24].

**Fig. 5:**
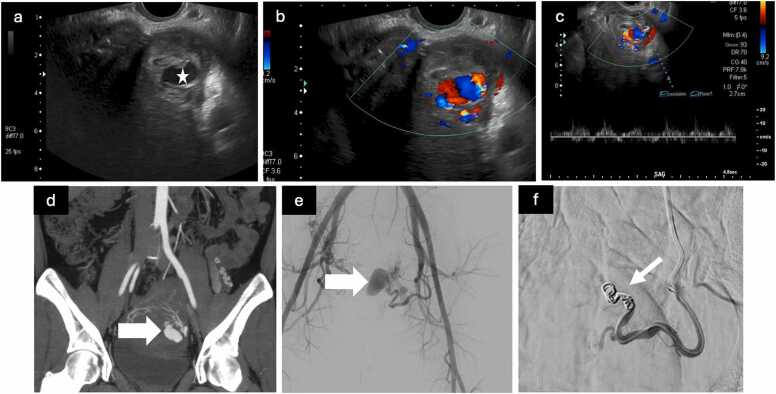
30 year– old female post C-section day 10 with postpartum heavy hemorrhage, tachycardia, and hypotension requiring blood transfusion. Transvaginal ultrasound (a) gray scale displays heterogenous myometrium with a central anechoic lesion (star) (b, c) Doppler interrogation shows localized myometrial vascularity with bidirectional flow pattern; “yin-yang” or “Pepsi” sign (d) Coronal CT pelvis maximum intensity projection (MIP) image and (e) Digital subtraction angiography image demonstrate a left uterine artery branch pseudoaneurysm (bold arrows) with (e) coil embolization (thin arrow). DIAGNOSIS: Uterine artery pseudoaneurysm.

Transvaginal (or transabdominal) ultrasound with color Doppler is the first diagnostic modality of choice. On grayscale, UAP typically appears as an anechoic or hypoechoic (or heterogeneous) lesion within or adjacent to the myometrium; on Doppler, the hallmarks are the so-called “yin-yang” sign (representing swirling bidirectional flow) and a “to-and-fro” spectral waveform at the neck of the pseudoaneurysm - this combination is considered pathognomonic and has reported diagnostic sensitivity up to ~ 95% [22]. If ultrasound findings are equivocal, the patient is haemodynamically unstable, or further vascular mapping is required (e.g., pre-embolisation planning), contrast-enhanced CT angiography (CTA) (or MR angiography) is indicated. CTA provides precise anatomical localisation, identifies the feeding artery, delineates the size and extent of the pseudoaneurysm (e.g., intramural vs exophytic), and detects active extravasation or associated haematoma - essential information for interventional radiology or surgical management [25]. Given its unpredictable natural history, UAPs require prompt diagnosis and timely management, commonly via selective uterine artery embolisation (UAE), which preserves fertility and avoids hysterectomy. Delay in diagnosis may lead to life-threatening haemorrhage, increased morbidity, and mortality (see Fig. 5).

### 1.7 Acquired uterine arteriovenous malformation (AVM)

**Fig. 6:**
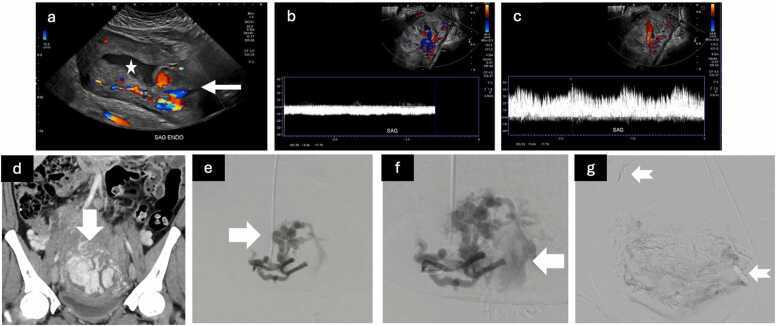
30-year-old female postpartum day 16 with complicated endometritis, new intense lower abdominal pain and postpartum hemorrhage. Doppler ultrasound shows (a) enlarged uterus with endometrial cavity filled with fluid (star) and a multilobulated vascular structure with turbulent flow (thin arrow), (b) venous flow and (c) low resistance high velocity arterial flow (d) contrast enhanced CT pelvis coronal image shows a multilobulated tortuous and enlarged uterine vascular lesion with early venous filling without active hemorrhage compatible with uterine arteriovenous malformation (bold arrow). Digital subtraction angiography selective uterine artery injection (e) outlines the uterine arteriovenous malformation (bold arrow) supplied by the left uterine artery (f) early venous filling during late arterial phase (bold arrow) (f) embolized feeding uterine artery and vein (notched arrows). DIAGNOSIS: Arteriovenous malformation.

Acquired uterine arteriovenous malformations (AVMs) are rare, likely underdiagnosed causes of secondary PPH. They are thought to arise after uterine trauma - most commonly caesarean section or evacuation of a scar ectopic pregnancy - when necrosed chorionic villi and placental tissue allow uterine venous sinuses to become incorporated into myometrial scars, creating abnormal arteriovenous shunts within the uterine wall [26]. Clinically, acquired uterine AVMs typically present in the delayed postpartum period with severe or recurrent haemorrhage, sometimes accompanied by lower abdominal or pelvic pain and a palpable or imaging-detected pelvic mass representing markedly hypervascular myometrium [27]. Because they are uncommon and can mimic retained products of conception or gestational trophoblastic disease, a high index of suspicion is required, particularly in women with recent uterine surgery or instrumentation. On ultrasound, AVMs appear as ill-defined, heterogeneous myometrial lesions with multiple serpiginous, tubular spaces demonstrating high-velocity, low-resistance flow on colour and spectral Doppler; CT or CT angiography shows a tangle of enhancing vessels within the myometrium, early venous filling, and sometimes associated haematoma, findings that guide planning for uterine artery embolization as a fertility-preserving treatment option [28] (see Fig. 6).

### 1.8 Role of interventional radiology

**Fig. 7:**
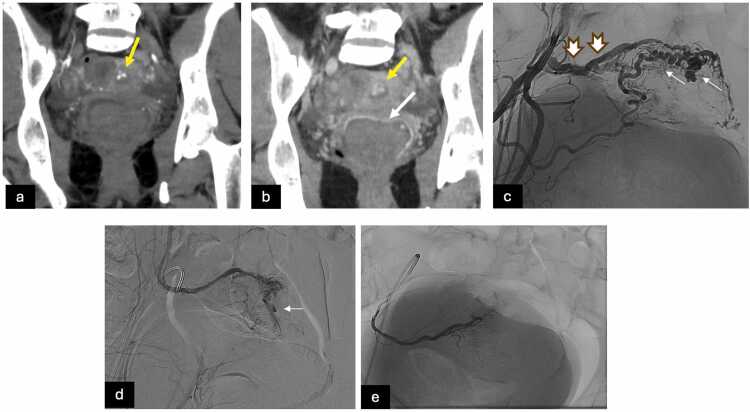
Status post stillbirth at 30 weeks. Complex recovery including retained products of conception 6 months post birth presented with heavy vaginal bleeding a) Coronal CT Angiogram showing pseudoaneurysm in the cavity and clot in the vaginal vault (yellow Arrow) b) Portovenous Phase showing active bleed into cavity (yellow arrow) and contrast extending around the existing clot in the vault (white arrow) c) Angiogram of the right uterine artery (white arrows) shows venous fistula formation with uterine vein (arrow heads) d) Late phase of the angiogram also shows active bleeding (white arrow) from the left side of the uterine artery e) Particle embolization performed using PVA (polyvinyl alcohol). Diagnosis: Pseuodaneurysm and venous fistula.

Uterine artery embolisation (UAE) involves selective catheterisation and embolisation of bleeding points in PPH to achieve haemostasis. Embolic materials include particles (250 microns and larger), Gelfoam, coils, plugs or liquid embolics (Cyanoacrylate, Onyx, Squid) [29]. It has been shown to be a safe and effective alternative to surgical treatment and has been shown to be successful in 80–90% of patients, offering a fertility-preserving alternative to hysterectomy and is increasingly gaining popularity. In addition, UAE does not increase gynaecological symptoms or cause sexual dysfunction [30] (see Fig. 7).

### 1.9 Retained products of conception

Retained products of conception (RPOC) refers to placental and/or foetal tissue that remains in the uterine cavity after birth, termination of pregnancy or miscarriage. It frequently presents with irregular or continuous vaginal bleeding and abdominal or pelvic pain with or without fever [31]. Histopathologically the tissue contains chorionic villi. It occurs in up to around 5% of pregnancies and can occur in any trimester, though the second trimester is most common. Risk factors for RPOC include abnormally invasive placenta, prior caesarean section, preterm birth and infection during labour (chorioamnionitis) [32].

For RPOC, the imaging modality of choice is ultrasound. On ultrasound, the findings are a thickened endometrial echo complex of over 10 mm or an echogenic intrauterine mass with increased vascularity. Based on colour Doppler findings, RPOC can be classified from 0 to 3: type 0 indicates no detectable vascularity within the thickened mass; type 1 indicates vascularity less than that of the myometrium; type 2 indicates vascularity nearly equal to the myometrium; and type 3 indicates vascularity greater than the myometrium. This is clinically relevant because type 0 RPOC is likely to resolve spontaneously without the need for intervention, whereas type 3 RPOC carries a higher risk of bleeding during dilatation and curettage [33].

The differential diagnosis for RPOC is a uterine AVM. While RPOC are endometrial in location and more common, a uterine AVM will be within the myometrium and are much less common [32], [34].

**Fig. 8:**
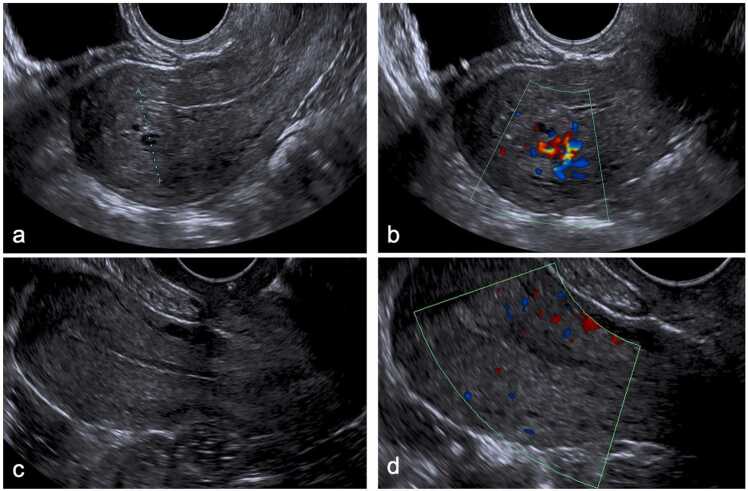
Retained products of conception: a) Ultrasound demonstrates echogenic mass (dotted line) within the endometrial cavity with b) Increased vascularity on Doppler. Post surgical evacuation of products of conception, return to c) Normal endometrial stripe and d) Normal vascularity. DIAGNOSIS: Retained products of conception.

*Learning point:* Doppler vascularity assessment is important for clinicians in deciding how to manage the patient. If vascularity of the mass is negative, it may be managed conservatively with serial follow up ultrasounds while a hypervascular mass may require surgical evacuation (see Fig. 8).

### 1.10 Endometritis

Endometritis refers to infection of the uterine decidua and occurs in up to 27% of caesarean births and 3% of vaginal births. Typically, this presents as fever, lower abdominal pain, and leukocytosis [35].

**Fig. 9:**
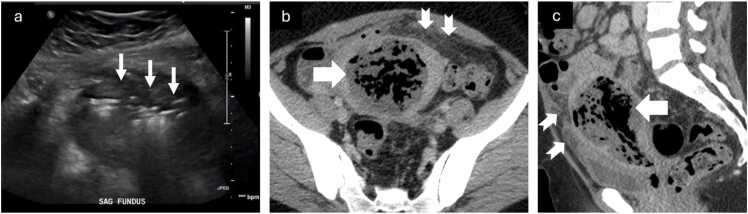
30-year-old female post caesarian section day 21 with postpartum haemorrhage, fever and ongoing purulent vaginal discharge. (a) Transabdominal ultrasound displays bulky uterus with multiple air foci in the endometrial cavity with distal shadowing and ring-down artifact (thin arrows). Contrast enhanced CT pelvis (b) axial and (c) sagittal images show gas and fluid distended endometrial cavity (bold arrows) with parametrial inflammation and fat stranding extending into the pro peritoneal fat (notched arrows). DIAGNOSIS: Endometritis.

It is predominantly a clinical diagnosis, as there is considerable overlap between normal and abnormal findings. However, there are some imaging findings which, in the presence of fever, malaise, sepsis, pelvic pain or foul-smelling lochia suggest endometritis. Ultrasound is the first line investigation and can show uterine enlargement. The endometrium can be normal or thickened and hypervascular with endometrial gas, seen as a ring down artefact. CT demonstrates similar findings but can additionally reveal parametrial fat stranding, abscess formation, septic thrombophlebitis, or infection extending along tissue planes (see Fig. 9) [36]. Caesarean section is a recognized risk factor for endometritis, and infection may track from the skin incision through the myometrium to the endometrial cavity [37] (see separate article).

Treatment is typically using antibiotics, with imaging reserved for complications or poor clinical response.

*Learning point:* Gas within the endometrial cavity may be physiological up to 3 weeks postpartum; correlation with clinical findings is essential, such as fever, pelvic pain or foul-smelling discharge [3], [36]

## 2 Myometrium

**Fig. 10:**
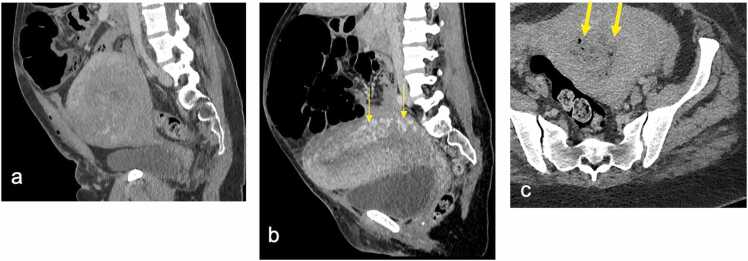
Normal appearances of the postpartum uterus on sagittal and axial CECT demonstrating a) Bulky myometrium b) Enhancing vascular myometrium c) Intrauterine gas on day 2 postpartum (yellow arrows), which resolved by day 12. DIAGNOSIS: Normal.

The postpartum myometrium can appear bulky and hypervascular as a normal finding [38] (see Fig. 10).

### 2.1 Pregnancy-related fibroid growth, degeneration, and acute haemorrhage

**Fig. 11:**
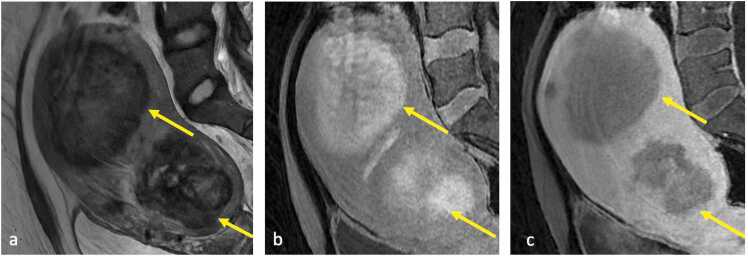
MRI findings of red degeneration of two uterine fibroids (yellow arrows). a) Sagittal T2 images demonstrates low signal in the fibroids b, c) Sagittal T1 fat saturated (FS) imaging pre and post contrast demonstrate high T1 signal in the fibroids that don’t enhance post gadolinium. DIAGNOSIS: Fibroid degeneration.

Leiomyomas (fibroids) are a common finding, occurring in up to 10% of pregnancies and increasing with advancing maternal age, and may undergo substantial change during pregnancy and the postpartum period [7]. Due to increased hormonal stimulation, vascular congestion, and rapid expansion of the gravid uterus, they may enlarge and outgrow their blood supply causing hyaline, cystic, myxoid, or red/haemorrhagic degeneration, or develop subcapsular venous congestion. MRI is the imaging modality of choice to confirm this and will typically demonstrate a high T1 and low T2 signal rim, corresponding to blood products in the peripheral vessels, and absence of enhancement on post-contrast imaging in red degeneration. In other degeneration types, fibroids demonstrate central necrosis, cystic areas, or myxoid signal patterns [39] (see Fig. 11)

Although most are asymptomatic, acute degeneration or rupture of surface vessels overlying a subserosal leiomyoma is rare but potentially life-threatening, particularly in women with a history of large fibroids or previous myomectomy. Sudden abdominal pressure changes or mechanical torsion may result in tearing of engorged surface vessels, causing haemoperitoneum. Clinically, patients present with acute abdominal/pelvic pain, signs of intra-abdominal bleeding, or haemodynamic instability, often without vaginal bleeding, prompting urgent imaging.

**Fig. 12:**
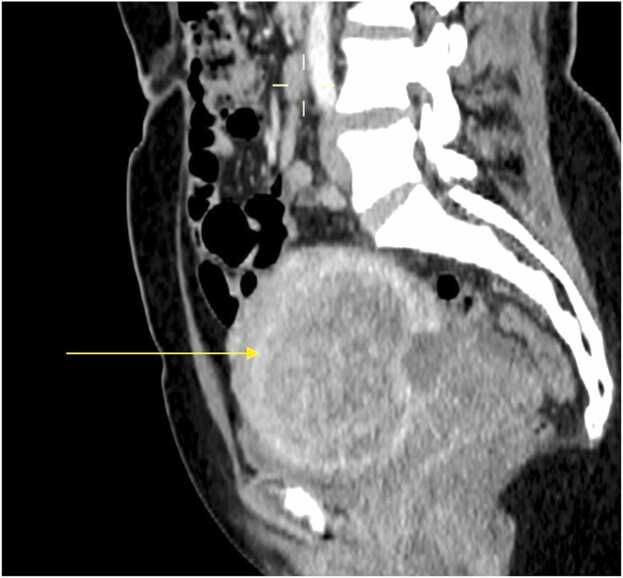
Sagittal CECT of a patient presenting with a 3-day history of pain demonstrates a dominant fibroid. The increased density (arrow) within the fibroid may represent enhancement or haemorrhage. DIAGNOSIS: Fibroid degeneration.

Ultrasound may show a heterogeneous leiomyoma with cystic or echogenic areas but is often limited in the acute setting. Contrast-enhanced CT is the modality of choice where ongoing bleeding is a concern, demonstrating active contrast extravasation, heterogeneous fibroid texture, or large intraperitoneal haematoma [40], [41] (see Fig. 12).

## 3 Pelvic and perineal haematomas

Intrapartum genital tract lacerations or vascular injury accounts for estimated 2–4% of postpartum haemorrhage [42]. They are anatomically categorised as infralevator or supralevator. Infralevator haematomas involve the vulva, vagina, perineum or ischiorectal fossa, and tend to present with external swelling, ecchymosis, or perineal pain [19]. In contrast, supralevator haematomas lie within the broad ligament or retroperitoneal space - these may be clinically occult, presenting instead with persistent postpartum bleeding, unexplained pelvic/abdominal pain, haemodynamic instability or shock, often without external signs.

Risk factors that predispose to postpartum haematoma formation include macrosomic infant, multiple gestations, primiparity, prolonged or precipitous labour, vulvovaginal varices, assisted vaginal birth, episiotomy or perineal incisions, and coagulopathy or bleeding disorders [19].

Imaging becomes essential in the following scenarios: suspicion of active haemorrhage (e.g., expanding swelling, ongoing bleeding, hypotension), clinical deterioration, or suspected superimposed infection of a haematoma. While ultrasound may suffice for superficial infralevator haematomas, contrast-enhanced CT (or CT angiography) is strongly indicated when a deep supralevator or retroperitoneal haematoma is suspected, for accurate delineation of haematoma extent, identification of contrast extravasation, and to guide possible interventional radiology or surgical management.

## 4 Bladder and ureters

Mild hydroureter can occur normally in pregnancy, and this may persist postpartum. Bladder or ureteric injuries are rare (occurring in less than 1%) but more likely in the presence of adhesions or repeat caesarean sections. Consider a bladder or ureteric injury particularly when the operation was complicated and when suspected, a CT cystogram is the investigation of choice for further evaluation [1]. Imaging findings on a urographic phase CT can show contrast extravasation, a non-contiguous ureter, hydroureter, and abnormal ureteric enhancement [43].

## 5 Abdominal wall

Divarication of recti is frequently seen and should not be mistaken for a fascial defect [9].

## 6 Ovarian veins

Physiological dilatation of ovarian veins commonly occurs in pregnancy, persisting postpartum [8].

Ovarian vein thrombosis (OVT) is a rare but important postpartum vascular complication seen in approximately 1 in 600–1 in 2000 births [25]. The vast majority of cases- about 80–90% - involve the right ovarian vein, with left-sided or bilateral involvement significantly less common [44]. The predilection for the right ovarian vein is likely multifactorial. Anatomically, the right ovarian vein is longer, often contains multiple incompetent valves, and drains directly into the inferior vena cava at an acute angle which predispose to stasis and thrombus formation. Additional risk factors include infection (e.g., endometritis or parametrial inflammation), pelvic surgery or instrumentation, and other conditions that augment thrombophilia [45].

**Fig. 13:**
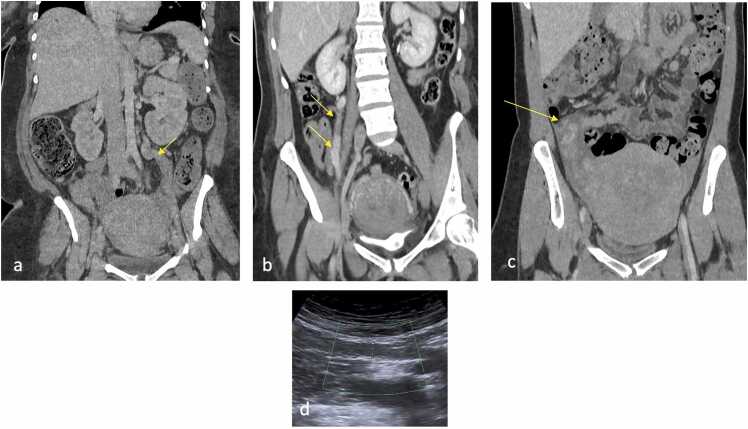
a, b): Coronal CECT demonstrating normal prominent ovarian veins. Note the smooth contours and no surrounding inflammatory stranding (arrows). DIAGNOSIS: Normal c) Coronal CECT demonstrated ovarian vein thrombosis. The right ovarian vein is engorged and inflamed with surrounding free fluid (arrow). d) Doppler US demonstrating absence of flow in the right ovarian vein consistent with thrombosis. DIAGNOSIS: Ovarian vein thrombosis.

Clinically, OVT typically presents in the first weeks postpartum with a triad of fever, lower abdominal/flank pain, and a tender pelvic or lower-quadrant mass; patients may also exhibit leukocytosis or signs of sepsis. Because presentation is often non-specific, OVT may be misdiagnosed as an acute abdominal conditions such as appendicitis or endometritis [44]. Doppler ultrasound is often the first-line modality which may reveal a dilated, non-compressible ovarian vein with intraluminal echogenic material and absent or diminished flow. However, sensitivity can be limited (e.g., due to overlying bowel gas or suboptimal acoustic window) [44]. When ultrasound is inconclusive, or when clinical suspicion remains high, contrast-enhanced CT or MR venography should be employed. On CT, typical findings include a dilated ovarian vein containing a low-attenuation thrombus, with absent contrast enhancement, often accompanied by perivascular fat stranding; in some cases, the thrombus may extend cephalad into the inferior vena cava or renal veins. Prompt diagnosis is crucial: untreated OVT may result in serious sequelae such as thrombus extension, pulmonary embolism, sepsis, or maternal morbidity, whereas timely anticoagulation (often combined with antibiotics if infection is suspected) leads to favourable outcomes (see Fig. 13).

## 7 Pelvic bones

**Fig. 14:**
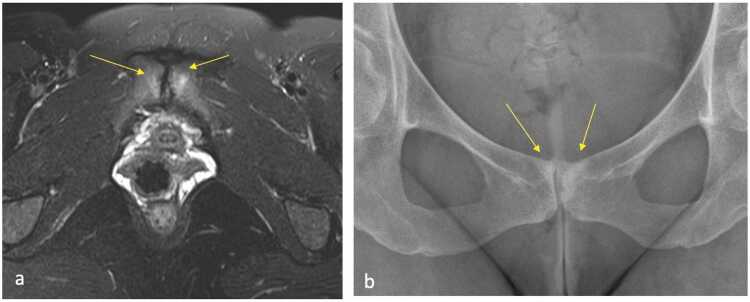
a) Axial T2 FS MRI performed 4 weeks after birth demonstrates bone marrow oedema of the pubic bone b) radiograph 1 year after birth demonstrates sclerosis consistent with osteitis pubis. DIAGNOSIS: Pubic bone marrow oedema.

Bone marrow oedema of the pubic symphysis is observed in up to 75% of women one week postpartum, following both vaginal and caesarean births. This usually resolves spontaneously and may evolve to become symptomatic or asymptomatic osteitis pubis. Persistent oedema and pain may indicate inflammatory arthritis [10] (see Fig. 14).

## CRediT authorship contribution statement

**Dick Prof Elizabeth:** Writing – review & editing, Writing – original draft, Conceptualization. **Taylor-Clarke Miss Marisa:** Writing – review & editing, Writing – original draft. **Qamar Dr Sadia Raheez:** Writing – review & editing, Writing – original draft. **Basilico Prof Raffaella:** Writing – review & editing. **Kashef Dr Elika:** Writing – review & editing, Writing – original draft. **Bharwani Dr Nishat:** Writing – review & editing, Writing – original draft. **Girija Agarwal:** Writing – review & editing, Writing – original draft, Conceptualization.

## Funding

No funding.

## Declaration of Competing Interest

R.B. is a Guest Editor for the VSI on non-traumatic body emergencies. The other authors have nothing to declare.
